# Corrigendum to ‘Modeling colorectal tumorigenesis using the organoids derived from conditionally immortalized mouse intestinal crypt cells (ciMICs)’ [Genes Dis 8 (2021) 814-826]

**DOI:** 10.1016/j.gendis.2023.02.001

**Published:** 2023-02-28

**Authors:** Xiaoxing Wu, Zhaoxia Li, Hongyu Zhang, Fang He, Min Qiao, Huaxiu Luo, Jing Zhang, Meng Zhang, Yukun Mao, William Wagstaff, Yongtao Zhang, Changchun Niu, Xia Zhao, Hao Wang, Linjuan Huang, Deyao Shi, Qing Liu, Na Ni, Kai Fu, Rex C. Haydon, Russell R. Reid, Hue H. Luu, Tong-Chuan He, Ziwei Wang, Houjie Liang, Bing-Qiang Zhang, Ning Wang

**Affiliations:** a Departments of Gastrointestinal Surgery, Medicine/Gastroenterology, And Obstetrics and Gynecology, The First Affiliated Hospital of Chongqing Medical University, Chongqing 400016, China; b Molecular Oncology Laboratory, Department of Orthopaedic Surgery and Rehabilitation Medicine, The University of Chicago Medical Center, Chicago, IL 606037, USA; c Department of Oncology, The PLA Rocket Force Characteristic Medical Center, Beijing 100088, China; d Departments of Burn & Plastic Surgery, West China Hospital of Sichuan University, Chengdu, Sichuan 610041, China; e Department of Orthopaedic Surgery, The First Affiliated Hospital of Guangzhou University of Chinese Medicine, Guangzhou, Guangdong 510405, China; f Departments of Orthopaedic Surgery, The Affiliated Zhongnan Hospital of Wuhan University, Wuhan, Hubei 430071, China; g Department of Orthopaedic Surgery, The Affiliated Hospital of Qingdao University, Qingdao, Shandong 266000, China; h Department of Clinical Laboratory Medicine, Chongqing General Hospital Affiliated with the University of Chinese Academy of Sciences, Chongqing 400013, China; i Ministry of Education Key Laboratory of Diagnostic Medicine, And the School of Laboratory Diagnostic Medicine, Chongqing Medical University, Chongqing 400016, China; j Department of Orthopaedics, Union Hospital of Tongji Medical College, Huazhong University of Science and Technology, Wuhan, Hubei 430022, China; k Department of Spine Surgery, Second Xiangya Hospital, Central South University, Changsha, Hunan 410008, China; l Department of Oncology and Southwest Cancer Center, Southwest Hospital, Army Medical University, Chongqing 400038, China

The authors regret that an image assembly (copy/paste) error in Figure 3D, in which the image for the organoid of “Primary MICs” group was erroneously duplicated with an image of primary MICs that was previously published. The corrected figure is shown below. As shown in the corrected Figure 3D, this error does not adversely impact the conclusion of the original work. The authors would like to apologise for any inconvenience caused.

**Corrected Figure 3**

**Figure 3:**
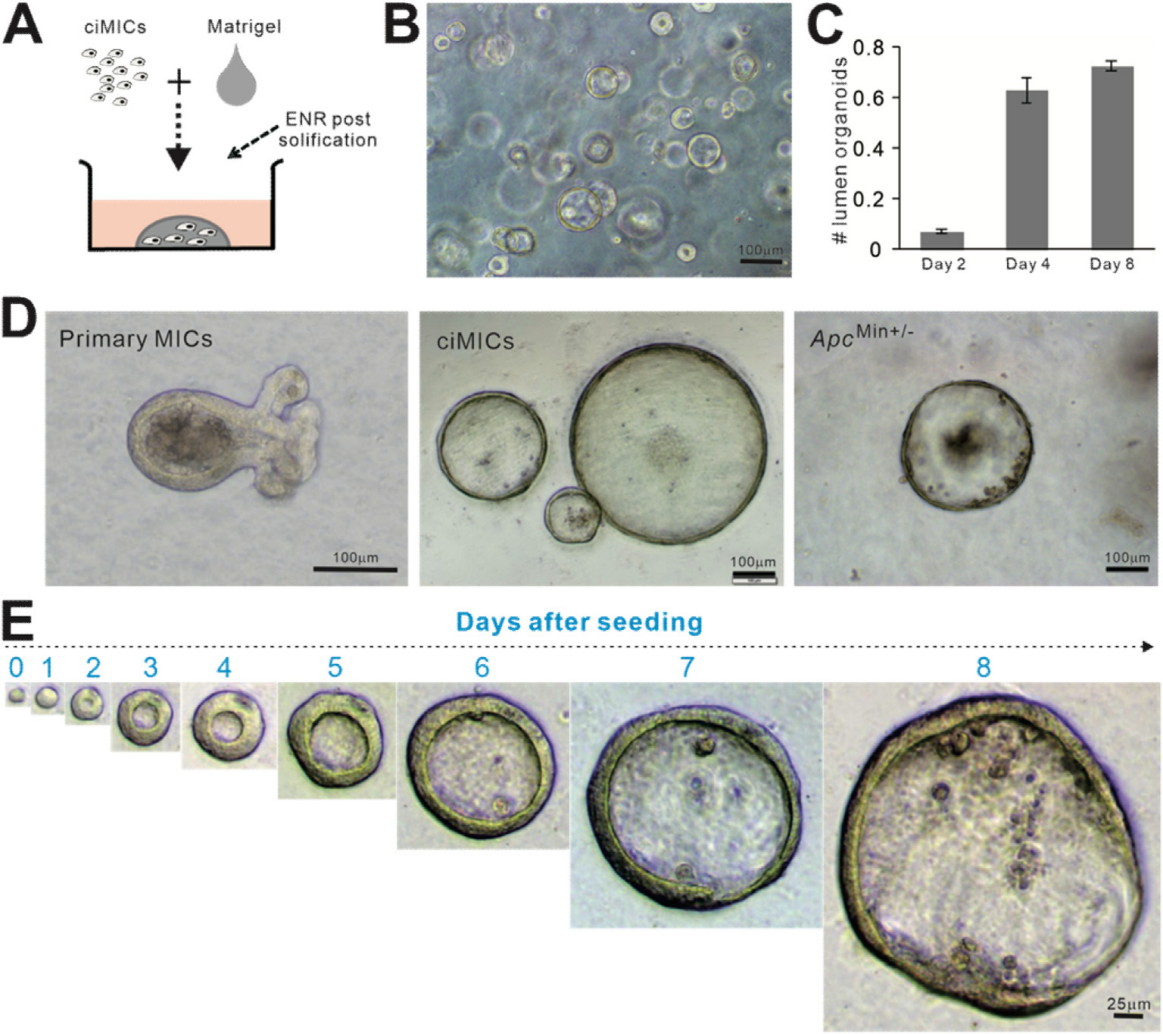
Development of three-dimensional crypt organoids from the ciMIC cells. **(A)** Schematic representation of sphere-cyst-like organoid formation in three-dimensional culture using ciMIC cells. **(B)** Morphological features of the organoids derived from ciMICs at 3 days after seeding in Matrigel. **(C)** Average rate of lumen-forming organoids at the indicated time points for ciMIC cells seeded in Matrigel. **(D)** Morphological features of the organoids derived from primary MICs, ciMICs and the MICs derived from *Apc*^*min+/−*^ mice. Representative images are shown. **(E)** Tracing the growth of organoid derived from ciMIC from a single cell in three-dimensional culture. Representative images are shown. Scale bar: B, D = 100 μm; E = 25 μm.

